# Randomized Embolization Trial for NeuroEndocrine Tumor Metastases to the Liver (RETNET): study protocol for a randomized controlled trial

**DOI:** 10.1186/s13063-018-2782-5

**Published:** 2018-07-17

**Authors:** James X. Chen, E. Paul Wileyto, Michael C. Soulen

**Affiliations:** 1 Division of Interventional Radiology, University of Pennsylvania, Philadelphia, PA USA; 2 Department of Epidemiology & Biostatistics, University of Pennsylvania, Philadelphia, PA USA; 3 Abramson Cancer Center, University of Pennsylvania, Philadelphia, PA USA; 4 Department of Radiology, Hospital of the University of Pennsylvania, 1 Silverstein, 3400 Spruce Street, Philadelphia, PA 19104 USA

**Keywords:** Neuroendocrine tumor, Embolization, Chemoembolization, Liver metastases

## Abstract

**Background:**

Neuroendocrine tumors (NETs) are the second most common gastrointestinal malignancy after colon cancer. Up to 90% of patients with NETs develop liver metastases, which are a major determinant of symptoms and survival. Current guidelines recommend embolotherapy for progressive or symptomatic NET liver metastases, but the optimal technique among bland embolization, lipiodol chemoembolization, and drug-eluting bead chemoembolization remains unknown and controversial.

**Methods/design:**

A prospective, open-label, multicenter randomized controlled trial will be conducted in patients with progressive or symptomatic unresectable NET liver metastases. Patients will be randomized to treatment with bland embolization, lipiodol chemoembolization, or drug-eluting microsphere chemoembolization, with 60 enrollees per arm. The primary endpoint will be hepatic progression-free survival (HPFS) following initial embolotherapy by RECIST criteria. The sample size is powered to detect an HR of 1.78 for HPFS following chemoembolization compared with bland embolization, which was estimated on the basis of existing retrospective studies. Secondary endpoints include overall progression-free survival, duration of symptom control, quality of life, rate of adverse events, and interval between embolotherapy cycles. Interim safety analyses will be performed at 10 and 30 patients per arm.

**Discussion:**

The RETNET trial is a prospective, multicenter randomized controlled trial designed to determine the optimal embolotherapy technique for NET liver metastases.

**Trial registration:**

ClinicalTrials.gov, NCT02724540. Registered on March 31, 2016.

**Electronic supplementary material:**

The online version of this article (10.1186/s13063-018-2782-5) contains supplementary material, which is available to authorized users.

## Background

Neuroendocrine tumors (NETs) are a heterogeneous family of neoplasms that arise from cells in the gastrointestinal (GI) tract, pancreas, and lung. With an estimated prevalence of > 100,000 in the United States and a steadily increasing incidence over the past quarter century [1], NETs have become the second most common GI malignancy after colon cancer. NET cancer biology has a propensity for the development of liver metastases, which occur in 40–90% of patients [2] and are a major determinant of symptoms and survival. Analysis of National Cancer Institute Surveillance, Epidemiology, and End Results registry data from 1973 to 2004 demonstrated that patients with localized, regional, or distant metastatic disease had median survivals of 223, 111, and 33 months, respectively [1]. With supportive care only, patients with unresectable liver metastases have a 5-year survival of 0–22%. Symptom control and survival outcomes improved in the late 1980s with the introduction of octreotide, a somatostatin analogue used to control hormone secretion-related symptoms. During the past three decades, there has also been widespread refinement and adoption of locoregional transarterial embolotherapy for treatment of NET liver metastases. By selectively embolizing hepatic arterial tumor feeders, metastases are cut off from their vascular supply, whereas the normal liver parenchyma retains viability via perfusion from the portal veins. Studies have demonstrated efficacy of these liver-directed embolotherapies for tumor growth reduction and NET symptom relief, with current 5-year survival rates of over 60% and response rates between 70% and 90% [3–13].

Liver-directed embolotherapy encompasses distinct techniques, including “bland” embolization (BE) using embolic particles instilled via the hepatic artery to cut off tumor blood supply and transarterial chemoembolization (TACE) using either an ethiodized oil emulsion of chemotherapeutic drugs (conventional lipiodol TACE, classical transarterial chemoembolization [cTACE]) or drugs loaded onto embolic microspheres (drug-eluting bead transarterial chemoembolization [DEB-TACE]). Current National Comprehensive Cancer Network, North American Neuroendocrine Tumor Society, and European Neuroendocrine Tumor Society guidelines support embolotherapy for symptomatic or progressive NET liver metastases on the basis of level IIB-3 evidence [14–18], but they offer no recommendation regarding available techniques.

Published studies comparing TACE with bland embolization (BE) have demonstrated heterogeneous and contradictory results, with some studies showing superior survival and/or safety outcomes for one technique [5, 6, 9] and others demonstrating equivalent outcomes [8, 19, 20]. One of the potential contributors to these varying results was ambiguity in the World Health Organization (WHO) 2000/2004 NET pathologic grading systems, which were used in many older studies [4, 6, 7, 9, 12, 21] and have since been replaced by the more objective, reproducible WHO 2010 system. In addition to uncertainty about survival outcomes among embolotherapy techniques, the comparative safety profiles and treatment toxicities also remain controversial. Two retrospective series have suggested higher risks for biliary complications after DEB-TACE [13, 22], but there remains no definitive comparison with lipiodol TACE or BE. The combination of an indeterminate evidence base and lack of specific guideline recommendations have contributed to wide variations in clinical practice, manifesting in equivalent use of available embolotherapy techniques for NET liver metastases in a survey of U.S. interventional radiologists [23].

Given the increasing incidence of NET and ongoing questions regarding an optimal treatment algorithm for NET liver metastases, there is a need for a prospective randomized controlled trial (RCT) to elucidate the optimal embolotherapy technique between transarterial BE and chemoembolization. This article presents the study protocol of a prospective, open-label, multicenter RCT using a parallel group assignment and noninferiority design. The trial aims to determine the optimal embolotherapy modality for NET liver metastases by comparing hepatic progression-free survival (HPFS), treatment toxicities, symptom control, and quality of life.

## Methods/design

### Hypothesis

The primary hypothesis of this study is that HPFS following TACE (lipiodol or DEB-TACE) will be nearly twice as durable as BE (HR, 1.78) for control of NET liver metastases. This will be tested against a null hypothesis of equality among embolization methods, without preconception of ranking.

### Study design

**Fig. 1:**
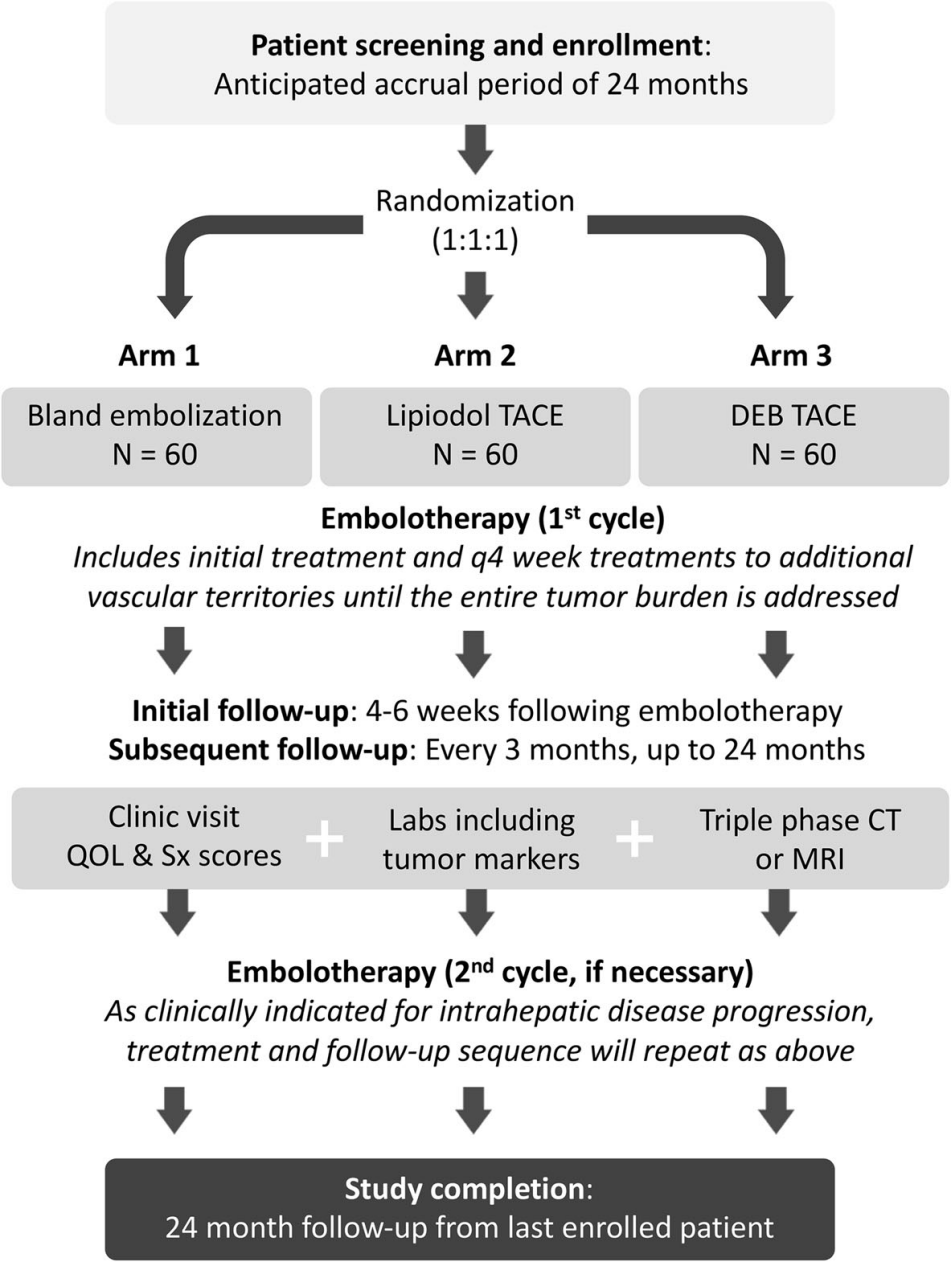
Study flowchart. *CT* Computed tomography, *MRI* Magnetic resonance imaging, *QOL* Quality of life, *Sx* symptom

This is an open-label, multicenter, randomized trial comparing three embolotherapy techniques for NET liver metastases: (1) BE, (2) cTACE, and (3) DEB-TACE. The study flowchart is shown in Fig. 1. Anticipated study sites include academic hospitals in the United States, Australia, Canada, France, and Argentina.

This trial will be conducted in accordance with the principles outlined in the Declaration of Helsinki and will follow the Consolidated Standards of Reporting Trials (CONSORT) statement. A Standard Protocol Items: Recommendations for Interventional Trials (SPIRIT) checklist is provided in Additional file 1.

### Study population

**Fig. 2:**
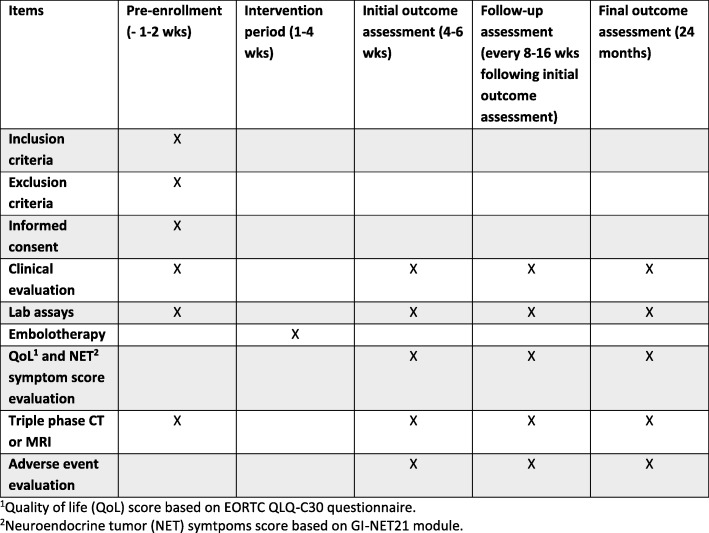
Standard Protocol Items: Recommendations for Interventional Trials (SPIRIT) figure. *CT* Computed tomography, *MRI* Magnetic resonance imaging

Eligible participants will have liver-dominant NET that is symptomatic, progressive, or with a liver tumor burden > 25% of the liver volume without need for documented progression. Patients with extrahepatic disease progression can be enrolled in the study if they have liver-dominant disease based on the assessment of a multidisciplinary NET care team. Concurrent treatment with somatostatin analogues is allowed. No concomitant radiation therapy, chemotherapy (e.g., capecitabine/temozolomide), biologic therapy (e.g., everolimus, sunitinib), or ablation is allowed. Patients also cannot receive these treatments until after the primary endpoint of hepatic progression is reached. All participants will be consented with an institutional review board (IRB)-approved, site-specific informed consent form prior to undergoing any study-related procedures. Detailed inclusion and exclusion criteria are shown in Table 1. A SPIRIT figure is shown in Fig. 2, and a SPIRIT checklist is available in Additional file 1. Patients may be withdrawn from the study for failure to adhere to protocol requirements or withdrawal of consent.

**Table 1:** RETNET trial inclusion and exclusion criteria

Inclusion criteria	Exclusion criteria
• Participants aged 18 years and older • Biopsy-proven neuroendocrine tumor with tumor burden dominant in the liver • Measurable metastasis to liver with at least one dimension ≥1.0 cm. Known extrahepatic disease should be limited to lymph nodes of < 2 cm and/or any bone metastases. • Liver tumor burden ≤ 70% of the total liver volume by visual estimate • Not a candidate for surgical resection owing to unresectability, anatomy, anesthesia risk, patient preference • Symptoms uncontrolled by somatostatin analogues OR morphologically progressive tumor by RECIST 1.1 criteria in the liver OR baseline tumor burden > 25% of the liver volume • No plans for the patient to receive other concomitant therapy while on this protocol treatment (other than octreotide or bisphosphonate therapy) • Performance status 0–2 on Zubrod performance scale • Serum creatinine ≤ 2.0 mg/dl • Serum bilirubin ≤2.0 mg/dl • Serum albumin ≥3.0 g/dl • Platelet count ≥ 50,000/μl (corrected if needed) • INR ≤ 1.5 (corrected if needed) • All patients must be informed of the investigational nature of this study and must sign a study-specific informed consent form in accordance with institutional and federal guidelines prior to study entry.	• Pregnant or lactating women may not participate, owing to the embryotoxic effects of protocol treatment. • Women/men of reproductive potential may not participate unless they have agreed to use an effective contraceptive method. • Prior hepatic arterial therapy or hepatic radiation therapy. Prior surgical resection or ablation of liver metastases is acceptable. Patients must be at least 1 month beyond prior chemotherapy, PRRT, ablation, or surgery and must have recovered from all therapy-associated toxicities. • Active infection (symptomatic bacterial and fungal infection, newly diagnosed and/or requiring treatment) • Choledochoenteric anastomosis, transpapillary biliary stent, or sphincterotomy of duodenal papilla • Absolute contraindication to intravenous iodinated contrast agent (history of significant previous contrast agent reaction not mitigated by appropriate premedication) • Allergy to doxorubicin • Contraindications to arteriography and selective visceral catheterization: ○ Severe allergy or intolerance to contrast media, narcotics, sedatives, or atropine * ○* Bleeding diathesis not correctable by usual forms of therapy * ○* Severe peripheral vascular disease precluding catheterization • Contraindications to hepatic artery embolization: * ○* Portal vein occlusion without hepatopedal collateral flow demonstrated by angiography or portal hypertension with hepatofugal flow * ○* Hepatic encephalopathy

*Abbreviations: INR* International normalized ratio, *PRRT* Peptide receptor radionuclide therapy, *RECIST* Response Evaluation Criteria in Solid Tumors, *RETNET* Randomized Embolization Trial for NeuroEndocrine Tumor Metastases to the Liver

### Investigations and interventions

#### Baseline evaluation

Clinical assessment, patient-reported NET quality of life (European Organization for Research and Treatment of Cancer [EORTC] QLQ-C30 and G.I.NET21 questionnaires) and Carcinoid Symptom Severity Score [24], screening laboratory studies (complete blood count, partial thromboplastin time, internal normalized ratio, comprehensive metabolic profile, tumor markers including chromogranin A), and imaging (abdominal computed tomography [CT] or magnetic resonance imaging [MRI], chest CT) will be performed within 30 days of initial embolotherapy. Patients with tumors without available histologic grading (WHO 2010) must undergo percutaneous biopsy to determine tumor grade. Once the eligibility data forms are completed in the ORACLE database (Oracle, Santa Clara, CA, USA) and baseline images are uploaded to the Imaging Core and undergo a quality control check, the treatment assignment is automatically generated via a built in block randomization program (*see* “Randomization” section below).

#### Embolotherapy technique

BE will be performed with lobar or segmental infusion of microspheres (100–500 μm) to an embolization endpoint of two- to five-heartbeat stasis. Conventional lipiodol TACE will be performed with lobar or segmental infusion of doxorubicin 50 mg, dissolved in 10 ml of diluted iodinated contrast agent, and emulsified with ethiodized oil, followed by embolization with 40–500-μm microspheres. DEB-TACE will be performed with lobar or segmental infusion of drug-eluting microspheres (100–300-μm or 300–500-μm microspheres loaded with doxorubicin per manufacturer’s instructions for use). For all treatment arms, embolotherapy to additional vascular territories will be performed every 4 weeks until the entire tumor burden is treated. The decision for lobar or segmental embolization and nuances of embolotherapy technique will be determined by operator preference.

#### Follow-up evaluation

Follow-up laboratory assays will be performed 3–3.5 weeks after the first embolotherapy session. If the patient remains eligible for further therapy, additional segmental or lobar embolization will be administered every 4 weeks until the entire disease area is adequately addressed. A maximum of four treatments can be performed per treatment cycle.

After the final embolotherapy session of the treatment cycle, serial clinic visits, QOL and NET symptom scores, laboratory assays including tumor marker(s), and triple-phase CT or MRI will be performed at 4–6 weeks posttreatment and then every 3 months ± 4 weeks (Fig. 1). If the patient remains within the eligibility criteria, repeat embolotherapy cycles can be performed when clinically indicated for intrahepatic progression. The second embolotherapy cycle will resume as described above. Total follow-up duration for each enrollee is at least 24 months. After this point, patients without disease progression will continue to receive clinical and imaging follow-up per institutional standard of care for the remaining duration of the trial.

### Study objectives

#### Primary endpoint

The primary endpoint is HPFS from initial embolotherapy by Response Evaluation Criteria in Solid Tumors (RECIST) 1.0 criteria, as determined by blinded central review of follow-up imaging. Because all of the embolization techniques are in common clinical use, there will be no formal interim efficacy analysis.

#### Secondary endpoints

Secondary endpoints include comparisons of the following:

- Time interval between embolotherapy cycles among embolotherapy techniques
- NET symptom-free interval using the Carcinoid Symptom Severity Score among embolotherapy techniques
- Patient-reported quality of life (EORTC QLQ-C30) and NET symptom (G.I.NET21) scores among embolotherapy techniques
- Toxicity and adverse events among embolotherapy techniques
- Overall progression-free survival (PFS) by RECIST 1.1 criteria and duration of symptom control between gut and pancreatic NET, as well as among tumor grades
- Biomarkers (imaging, laboratory, and/or symptom) for treatment effect in all treatment arms

#### Primary safety endpoint

According to the Society of Interventional Radiology quality improvement guidelines for embolization and chemoembolization, major hepatic complications (biloma, abscess, liver failure) occur following approximately 6% of procedures, and the performance threshold is set at 8% [25]. The overall rate of any complication is 10%, with a performance threshold of 15%. All arms will be monitored for serious adverse events (SAEs) exceeding published thresholds. SAEs are classified as any event that is fatal; life-threatening; requires or prolongs hospital stay; results in persistent or significant disability, congenital anomaly, or birth defect; or an important medical event. Adverse events will also be classified using Common Terminology Criteria for Adverse Events (CTCAE) grading. Liver function test elevations of CTCAE grade 3 or lower and any clinical symptoms attributable to postembolization syndrome will not be reported unless they meet a criterion. All toxicities/events at or above CTCAE grade 4 will be reported. SAEs will be reported to the study sponsor by telephone and study form within 24 hours of the event. Interim safety analyses will be performed by an independent safety monitoring committee, which is composed of three interventional oncologists from institutions not involved in the trial, following accrual of 10 and 30 patients in each arm. A 20% rate of SAEs at these interim analyses will result in closure of the corresponding treatment arm.

### Statistical considerations

#### Sample size calculation

Based on available retrospective data, median HPFS following BE is estimated at 11 months and cTACE at 20 months [9], corresponding to an HR of approximately 1.8. The effect of cTACE and DEB-TACE is presumed to be equivalent. Assuming a two-sided type I error rate of 10%, 60 participants per arm will provide at least 92% power for the overall log-rank test and 87% power for pairwise tests.

### Randomization

The study will enroll 180 patients, who will be randomized in a 1:1:1 ratio to the three treatment arms. Randomization will be performed centrally and stratified by institution, using blocks of randomly selected sizes (3, 6, or 9). Patients with symptomatic but not progressive liver disease will be monitored during the accrual phase to ensure balanced allocation between treatment arms, with adjustment as necessary.

### Trial time scale

Preliminary experience from the pilot institution suggests that approximately six participants per site can be enrolled in 1 year. With at least 16 sites anticipated to participate in the study, a 24-month accrual period would result in approximately 180 enrollees. This provides an adequate margin for the target sample size of 194 patients, assuming a dropout rate of 8%. Follow-up is a minimum of 24 months.

### Statistical analysis

Our analysis will be aimed at identifying any embolotherapy arm that is significantly inferior (or superior) to the others based on comparison of the primary outcome. Our null hypothesis assumes that the arms are equivalent, without preconceived ranking. The test will use a studentized range approach developed for phase II screening trials using survival data [26, 27]. We will estimate the survival hazard rate in each treatment arm and compute the range of log-hazard rates (largest minus smallest). We will then compare the observed range to the distribution of the range under the null hypothesis. If the observed range exceeds the 95th percentile of its null distribution, any group whose hazard rate differs by more than this critical value from the group with the lowest PFS hazard will be declared significantly worse.

For secondary outcomes including symptom relief interval, time to reembolization, overall PFS, and PFS for subsets of the study sample, we will estimate the survival distribution using the Kaplan-Meier method. Comparisons among groups will be performed using log-rank tests, and survival regression models will be developed to estimate treatment effects, adjusted for baseline variables that may affect treatment outcomes.

For continuous outcomes (e.g., quality of life and symptom scores), summary statistics (mean, SD, 95% CI) will be assessed for each of the study arms, and comparisons among arms will be performed using one-way analysis of variance and *t* tests. Regression models will be used to estimate treatment effects adjusted for baseline variables that may affect treatment outcome. Binary variables (e.g., biomarkers) will be tabulated by treatment arm and compared using the chi-square test. Logistic regression models will be used to estimate adjusted effects.

### Data management and monitoring

Data management will be handled by the Clinical Research Computing Unit at the University of Pennsylvania. Study data will be entered in an ORACLE database. Data entry will be performed by study coordinators and investigators at each of the sites.

Triphasic abdominal CT and MRI images will be uploaded to the Imaging Core at the American College of Radiology in Philadelphia and undergo central review by independent, blinded diagnostic radiologists unaffiliated with the participating institutions.

For patients who withdraw following embolotherapy, reasonable efforts will be made to track adverse effects, follow-up imaging results, and survival through the medical record at the treating institution by their treating physician.

Data quality checks have been built into the ORACLE data collection instruments. All database information will be encrypted, and de-identified data will be used for analysis. All investigators will permit study-related monitoring, audits, and inspections by the ethics committee, IRB, sponsor, government regulatory bodies, and university compliance and quality assurance groups of all study-related documents (e.g., source documents, regulatory documents, data collection instruments, study data). Remote audits of 5% of the data from each site will be perfumed at 50% accrual and at completion of accrual.

### Ethical considerations and dissemination

The study protocol and any amendments will be submitted to a properly constituted independent ethics committee or IRB, in agreement with local legal prescriptions, for formal approval of the study conduct. The formal consent of a subject, using the ethics committee/IRB-approved consent form, must be obtained before that subject undergoes any study procedure. The consent form must be signed by the subject or legally acceptable surrogate and by the investigator-designated research professional. Unanticipated problems posing risks to subjects or others as noted above will be reported to the sponsor institution IRB.

Each subject is assigned a unique patient identification (PID) number in the ORACLE database. All research data and images are de-identified at the source and associated with the PID number.

The principal investigator is responsible for publication of the results of this study in whole or in part. Neither the complete nor any part of the results of the study carried out under this protocol, nor any of the information provided by the sponsor for the purposes of performing the study, will be published or passed on to any third party without the consent of the study sponsor. Any investigator involved with this study is obligated to provide the sponsor with complete test results and all data derived from the study.

## Discussion

Despite several decades of experience with embolotherapy for NET liver metastases, there remains uncertainty regarding the optimal treatment modality and a paucity of level I evidence. Compared with other primary and metastatic liver malignancies, NET liver metastases have superior oncologic outcomes with a median survival of 18–65 months from initial embolotherapy [4–6, 9, 10, 12, 20, 21]. The relatively indolent disease course and multimodality treatment approach have unique implications for determining the optimal liver-directed embolotherapy. The chosen technique should ideally not only achieve superior survival and local disease control but also have tolerable toxicity when repeated and used in combination with other therapies. The existing evidence base does not provide a clear answer regarding which embolotherapy best satisfies these criteria, reflected in lack of guideline recommendations specifying a specific embolotherapy technique [14–18].

The Randomized Embolization Trial for NeuroEndocrine Tumor Metastases to the Liver (RETNET) will be the first multicenter prospective RCT aimed at elucidating these questions regarding optimal embolotherapy technique. The results of this study will help refine the role of embolotherapy for NET liver metastases by determining which, if any, embolotherapy technique confers superior HPFS and/or safety profile between BE, lipiodol TACE, and DEB-TACE.

### Trial status

Patient enrollment started in March 2017 and was ongoing at the time the manuscript was submitted. Completion of accrual is expected to take 24 months.

## Additional file

Additional file 1: Standard Protocol Items: Recommendations for Interventional Trials (SPIRIT) 2013 checklist: recommended items to address in a clinical trial protocol and related documents, in addition to IRB approval letter. (DOCX 45 kb)

## Abbreviations

BE: Bland embolization
CONSORT: Consolidated Standards of Reporting Trials
CT: Computed tomography
cTACE: Classical transarterial chemoembolization
CTCAE: Common Terminology Criteria for Adverse Events
DEB-TACE: Drug-eluting bead transarterial chemoembolization
EORTC: European Organization for Research and Treatment of Cancer
GI: Gastrointestinal
HPFS: Hepatic progression-free survival
INR: International normalized ratio
IRB: Institutional review board
MRI: Magnetic resonance imaging
NET: Neuroendocrine tumor
PFS: Progression-free survival
PID: Patient identification
PRRT: Peptide receptor radionuclide therapy
QOL: Quality of life
RCT: Randomized controlled trial
RECIST: Response Evaluation Criteria in Solid Tumors
RETNET: Randomized Embolization Trial for NeuroEndocrine Tumor Metastases to the Liver
SAE: Serious adverse events
SPIRIT: Standard Protocol Items: Recommendations for Interventional Trials
TACE: Transarterial chemoembolization
WHO: World Health Organization
